# Retraction Note to: Increased neuroinflammatory and arachidonic acid cascade markers, and reduced synaptic proteins, in brain of HIV-1 transgenic rats

**DOI:** 10.1186/s12974-017-0872-z

**Published:** 2017-05-09

**Authors:** Jagadeesh Sridhara Rao, Hyung-Wook Kim, Matthew Kellom, Dede Greenstein, Mei Chen, Andrew David Kraft, Gaylia Jean Harry, Stanley Isaac Rapoport, Mireille Basselin

**Affiliations:** 1 Brain Physiology and Metabolism Section, National Institute on Aging, Bethesda, MD 20892 USA; 2 National Institute of Mental Health, National Institutes of Health, Bethesda, MD 20892 USA; 3 Laboratory of Toxicology and Pharmacology, National Institute of Environmental Health Sciences, National Institutes of Health, Research Triangle Park, NC 27709 USA

## Retraction Note

This article [1] has been retracted by the editor because author Stanley I Rapoport alerted the editor, and the National Institutes of Health subsequently confirmed, that the data represented by Figs. 1, 4, 5, 6 and 7 were falsified. Stanley I Rapoport, Andrew David Kraft, Gaylia Jean Harry, Matthew Kellom, Hyung-Wook Kim and Dede Greenstein support this retraction. The other authors have not responded to our correspondence with them about the retraction of their article.
